# Searching Nanoplastics: From Sampling to Sample Processing

**DOI:** 10.3390/polym13213658

**Published:** 2021-10-23

**Authors:** Marina Cerasa, Simona Teodori, Loris Pietrelli

**Affiliations:** 1 National Research Council of Italy-Institute of Atmospheric Pollution Research (CNR-IIA), Via Salaria km 29,300, 00015 Monterotondo, Italy; marina.cerasa@iia.cnr.it; 2 Chemistry Department, Sapienza University of Rome, Piazzale Aldo Moro, 00185 Rome, Italy; simona.teodori@uniroma1.it

**Keywords:** nanoplastics, microplastics, sampling, sample treatment

## Abstract

Nanoplastics (NPs) are considered emerging pollutants, namely unregulated contaminants whose toxic effect on humans and the environment has been demonstrated or suspected. They are the result of the physical fragmentation of the plastics that over time reach smaller dimensions (<100 nm). The issues related to the characterization and quantification of NPs in the environmental matrices are mainly related to the infinitepsimal size, to the fact that they are found in bulk, and to the different physico-chemical forms in which the same polymer can evolve over time by degradation. To deal with the study of a new class of pollutants it is necessary to assess the entire analytical method, carefully considering every single step (sampling, cleanup, qualitative, and quantitative analysis) starting from the validation method in the laboratory. This paper reviews the analytical method steps, focusing on the first ones, which the current literature often underestimates: laboratory tests, sampling, and sample processing; in fact, most errors and the quality of the analyses often depend on them. In addition, all newly introduced sample processing methods were examined.

## 1. Introduction

Plastic is widely used because of its low cost, versatility, and durability. The global production of plastics has increased significantly, rising from 1.5 million metric tons in 1950 to 359 million metric tons in 2020 [1]. Considering the short “in-use” lifetime, packaging is considered one of the major sources of waste worldwide [2]. Prior to 1980, 100% of plastic waste was delivered to landfill or lost into the environment. In Europe in 2018, 29.1 million plastic (47.1% of the total EU production) was collected and of these 42% was converted into energy, 32.5% recycled, and 24.9% landfilled [2,3,4].

Describing plastic waste generation by polymer types, the most widespread are non-biodegradable such as polyethylene (PE), polypropylene (PP), polyethylene terephthalate (PET), polyvinyl chloride (PVC), and polystyrene (PS). Considering all the polymers produced by the industries, PE, PP, and PET are widely recycled [2]. Due to accidents and inadequate consumer behavior mismanaged waste plastic is released to air, soil, and water systems increasing the dispersion and accumulation in the environment. Once in the environment, depending on the exposure to solar radiation, mechanical forces or interactions with organisms, plastics can slowly degrade [5,6], therefore fragmentation into smaller particles commonly known as microplastics (MPs) and nanoplastics (NPs) can take place. Within the scientific community there is still no consensus on the definition of MPs and NPs. Some authors considered MPs as particles in a range between 1 µm and 5 mm and NPs in a range between 1 nm and 1 µm; [7,8,9,10,11] others defined NP as <100 nm particles size, [12,13,14] based on the European Commission definition of the nano-material size that is 1–100 nm [15]. Schwaferts et al. 2019, for example, considered MP size range from 1 µm to 5 mm, subµ-plastic ranges from 100 nm to 1 µm and NP ranges from 1 nm to 100 nm [12]. To date, commonly used categorization system is based on size using the prefixes of mega, macro, meso, micro, and nano. MPs and NPs are also distinguished according to their origin, into “primary”, when they are produced to be of microscopic dimensions, (plastic pellets serving as precursor for manufactured plastic products, industrial cleaners and personal care products such as toothpaste, facial and body scrubs, etc.,) or “secondary”, resulting from degradation and fragmentation processes occurring in the environment. Fibers from synthetic clothes are also considered as secondary MPs [10,16,17].

**Figure 1:**
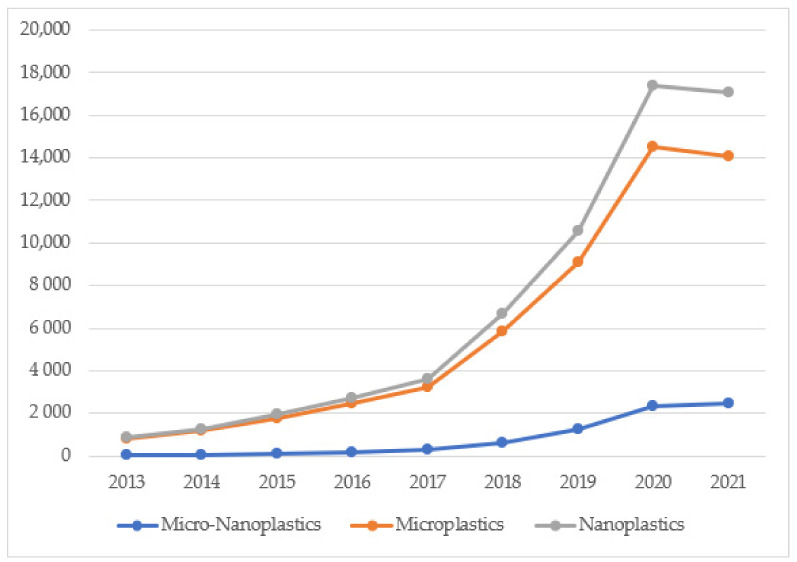
Trend of the number of publications on Microplastics and Nanoplastics topics from 2013 to August 2021. The survey was conducted between July–August 2021 using accessible online databases such as Web of Science, Scopus, Science Direct, and Scholar Google. Search criteria: microplastics, nanoplastics, water (title + abstract + key words).

A significant increase of scientific interest and public concern about the fate of MPs and NPs in the environment and their potential harmful effects can be deduced by the increase of paper published during the last 5 years (800%) as reported in Figure 1.

**Figure 2:**
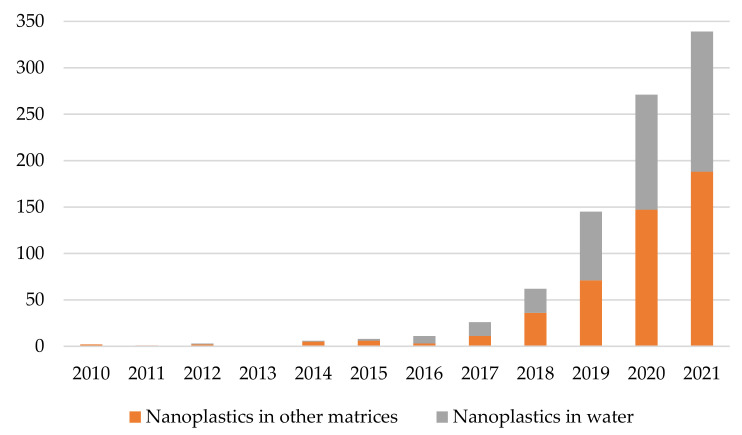
Trend of publications on Nanoplastics from 2010 to 2021. Comparison of studies focused on the water matrix compared to all the other matrices. Data collected from Scopus.

In particular the impact of plastic waste on marine and freshwater habitats appears to be one of the most studied phenomena and the cause of the greatest concerns especially for NPs (see Figure 2 and Supplementary Material Figure S1).

Both MPs and NPs can be ingested by a wide range of marine species [18,19]. This ingestion can result in physical damage such as obstruction or internal abrasions [20] or can greatly reduce stomach capacity, leading to false sense of satiation. This feeling of fullness can reduce dietary intake and this can cause growth inhibition [21,22,23], reduction of fertility [24,25], energy depletion [26,27]. Furthermore, MPs and NPs could release toxic additives incorporated into a plastic during manufacturing to improve its properties (e.g., brominated flame retardants like polybrominated diphenyl ethers (PDE) or phthalates used as plasticizers) [28] and could also adsorb on the surface several persistent organic pollutants (POPs) and heavy metals, which is due to their larger specific surface area and hydrophobic properties [29,30]. Later, the MPs and NPs can release those POPs to organisms upon ingestion [31,32,33] and could impact human health, therefore it is important to determine their fate and amount. Considering their size and interaction with the biota, NPs can be distinguished from MPs and, in particular, from the engineered nanoparticles [34], therefore an increase of difficulty to analyze NPs in the environmental matrices can be expected.

There are no specific methodologies for NPs and often the same techniques used for MPs are adapted.

To analyze MPs and NPs in the environment there are some important steps to be done such as: to determine the sampling points, to choose the sampling equipment according to the matrix that can be employed, the pretreatment and treatment of the sample (stabilization of the organic matter, reduction in the volume of sample, destruction of biofilm [35,36], collection of by products, and disposal of the sample in a safe way before the polymer characterization) and, finally, to choose the identification techniques (Table S1).

Water, sludge, air, or soil sampling is the first step toward the characterization and quantification of MPs and NPs but unfortunately there is still no standardized sampling method in particular in the wastewater treatment plants that is the final step of the anthropogenic water cycle and the link with the environment.

Some aspects cannot be underestimated when developing an analytical method and should be considered “validated” and before application on real samples. The technical parameters that must be met for the validation of a method are specificity, selectivity, accuracy, and precision, uncertainty, the limit of detection, the limit of quantification, robustness and recovery [37,38,39]. It can therefore be said that reliable values are obtained only when the quality assurance/quality control (QA/QC) is satisfied. The latter certainly includes the traceability of the method (which allows it to be reproduced by other operators as well), the inter-laboratory study, the uncertainty of the data (without which the results are only random values), the use of a validated method, and the use of standardized materials [40].

In this paper sampling, pre-treatment, and treatment methods for MPs and NPs of the environmental and anthropogenic matrices are summarized. In this context, this review focuses on the current information on the available methods concerning the whole analytical process from sample treatment to particle characterization in various matrices. The main purpose of the current review is to report the main key aspects of the most reliable techniques and discuss their advantages and limitations.

## 2. Simulated Samples-Laboratory Tests

As is often the case for most emerging contaminants (ECs), analytical methods developed and validated for other contaminants have been used up to now for the study of nanoplastics (NPs). It can be assessed that this class of ECs does not currently have a real analytical protocol for their identification and quantification in real matrices. There are very few scientific works that currently attempt the identification of polymers in real matrices, most of the literature in fact deals with in vitreous or spike tests in the laboratory [41].

Undoubtedly it is very difficult to develop a new method to determine which polymers, often degraded, their concentration, their size, morphology, and color in environmental matrices different from each other in origin and physical characteristics. In terms of DoE (design of experiment) to predict the outcome of the experiment necessary to identify the variables affecting the resulting data, the deep knowledge the habitat, foraging niche, etc., in other words the “environment”, of the sample site. Considering that certified MPs and NPs containing standard materials do not yet exist or they are not very widespread, the resulting data are often subject to variations, therefore statistical replicas are absolutely necessary. An interesting inter-laboratory comparison study on PET microparticles in water has been organized by the European Commission [42].

Each environmental matrix (sand, sludge, wastewater, drinking water, animal tissues, etc.,) requires a different procedure (i.e., procedure parameters) to separate the NMPs from the matrix and often no data regarding the “recovery rate” of the utilized method are reported. Following a first screening of the polymers contained in a matrix, it would be advisable to insert a known quantity of MPs and NPs to subsequently evaluate the effectiveness of the method.

Currently there are a few studies that have attempted the DoE approach for the study of NPs to develop the methodology that takes into account all the variables. In the work of Fraissinet et al. 2021 the DoE was adopted for the microwave extraction of MPs by evaluating (1) the digestion efficiency (% DE) of the organic matter and (2) the plastic recoveries (% PR) [43].

There are several works in which the need for the application of DoE is highlighted. Ogonowski et al. 2021 states that tests with both anthropogenic and natural microplastics are not representative and often the results contrast with those of other researchers. Food, water, etc., require periodic and constant checks that respect protocols and are representative of the population [44].

Here some difficulties that arise from the variables of the NPs compare to the MPs.

*Size, shape, and composition*: NPs can have primary origin if they enter the environment with nanometric dimensions whose size depends on its original specific use (e.g., cosmetics, ink for 3D printers) or secondary origin from the degradation of both macro (>5 mm) and microplastics (5 mm–1 µm). Physico-chemical characteristics such as surface area, ionic strength, conductivity, and reactivity are very different at the nanoscale compared to micro and macro plastics. For example, it has been verified that as the size of the plastic particle decreases, the biological reactivity increases. It is therefore essential to understand the burden of the availability of NPs and its biological impact on ecosystems [45]. Furthermore, in real samples a mix of polymers can be found and the composition, additives, and characteristic products of each polymer are difficult to distinguish from each other [46]. As the plastic size decreases, the contribution of organic matter/microbial increases influencing the transport, uptake, and accumulation pathways of NPs [34]. Furthermore, the nanoscale size limits chemical analysis techniques; all the techniques that are popular in MPs analysis have a limited application for NPs. There are much more suitable separation and analytical techniques typically applied to engineered nanomaterials (ENMs particle size between 1 and 100 nm) like cross-flow ultrafiltration, asymmetric flow field flow fractionation, nanoparticle tracking analysis, and dynamic light scattering [41,47].

*Aggregates*: The presence of NPs in the form of aggregates influences their transport, mobility, and resilience, as well as the mobility within different environmental compartments (atmosphere, water, soils) and consequently interaction with living organisms. The aggregated NPs will tend to settle while the free ones will be subject to long-distance transport. The aggregation of NPs depends on many factors such as UV rays, atmospheric agents, biodegradation, inorganic colloids, and organic matter. For example, this last factor causes an aggregation, given the larger size and the neutralization of the isolation point of the NPs, their tendency to enter biological membranes decreases [45]. Owing to the dominance of Brownian diffusion rather than sedimentation and buoyancy, nanoplastic heteroaggregates, are characterized by random distribution and movements compared to the MPs composed of the same material. This limits the application of density separation methods such as sedimentation and centrifugation that are more suitable for MPs. About the analytical approaches one of the main complication is the heteroaggregation that can interfere from the background material, carbon in particular [41,47]. Moreover, for the aggregates the similarity of the NPs is closer to that of the ENMs rather than to the MPs: they form heteroaggregates similar to microorganisms and natural organic matter [34]. *Aging*: all the factors previously mentioned (e.g., UV, biodegradation, atmospheric agents) alter the surface characteristics of NPs, generating many by-products that cannot be ignored [48]. Another variable linked to degradation is the matrix in which they are present, in fact we know that the polymer itself undergoes accelerated degradation in air rather than in water [49].

To date, one of the major problems is the absence of standardized materials for NPs, interference and environmental contamination (laboratory equipment, operators’ clothing, personal care, furniture, etc.,). In the work of Materić et al. [48] a semi-quantitative methodology is developed through a fingerprint of the characteristic ions and recognition in the library of polymers using the thermal-desorption proton-transfer-reaction mass spectrometer (TD-PTR-MS) starting from a methodology coming from previous studies [49,50]. Polymer reference materials and identification scores are provided in addition to the use of laboratory blanks before testing on real samples [51], the same method is then applied on real snow samples in the Alps [49]. This analytical growth approach today represents one of the few works in the scientific literature, including sludge from wastewater treatment plants [52].

Given the difficulty in characterizing an environmental mix of NPs, many works tend to perform instrumental analyses on individual polymers by identifying a fingerprint in the response and the same polymers called standards are then added to the environmental matrix [47]. This is the case of the TGA (thermo gravimetric analysis) extraction optimization study by varying the heating rate and the purge gas flow; before application on real samples, the repeatability of the method was evaluated [53].

Currently there are no isotopically labeled standards of NPs and it is not possible to apply the isotopic dilution method often used for most atmospheric pollutants. Tracers for NPs based on rare earths (Eu, used as an additive) have been hypothesized [46]. When it comes to standard materials, we refer to polymers purchased individually and crushed simply through grinders [54] or with more imaginative methods such as through stones and ultrasonic cycles [55], or purchased already at the nanometer scale [47].

*Sample contamination*: Unintentional contamination from the air or from the researchers’ synthetic clothing is a common problem that is believed to lead to an overestimation of MP fibers in environmental samples [56]. Polymeric materials should be avoided for instruments and setups to prevent systematic sample contamination. According to Schwafert et al. a blank value and a method recovery evaluation are essential to correct this kind of trivial errors as much as possible [12].

This problem is addressed in several scientific publications. During sampling as well as in the laboratory, in order to prevent contamination, it is recommended to take the following measures: (i) The use of cotton jackets instead of synthetic fleece during sampling and white cotton coats during sorting in the laboratory [8,57,58,59,60]; (ii) wash all equipment with ultrapure water before covering it with a clean sheet [8,57,58,59]; (iii) regularly clean the laboratory and work area; (iv) minimize the exposure time of samples to air, for example by storing them in covered Petri dishes [58]; (v) processing the samples inside a clean laminar flow hood [12,57].

In addition to these practices, contamination is also assessed using procedural blanks as a contamination control measure [51,53]. These blanks are processed in the same way as samples to evaluate other sources of contamination during sample processing and analysis. For example, to quantify airborne contamination in the laboratory during sample processing, some moist filter papers are placed in Petri dishes and exposed to the air inside the hood and on the laboratory benches during each instance of sample processing [58]. In another work, the dampened glass fiber (GF/C) filter paper was left open to the air both aboard the boat and in the laboratory at each stage of processing and screened for plastic contamination using an optical microscope [53]. Even more cautious has been the choice of some authors to limit or avoid movements in the laboratory (e.g., walking) when handling open samples (e.g., loading) [49]. Many of these errors in the use of standard polymers are overcome by using specific fluorescent dyes through which the polymers are labeled [47,55]. The latter is often associated with fluorescence quantification techniques.

*Representativeness*: In recent years, the scientific effort to develop the analytical procedures for NPs characterization has been increasing. For example, through the study of extraction efficiency, as in the work of Díaz-Jaramillo et al., where a known concentration of MPs is spiked in a natural clean sand [54]. This study includes laboratory blanks, replicates and the identification of false positives by meeting the criteria of selectivity, evaluation of the matrix effect, repeatability, and providing an error on the procedure [49].

Recently a quantitative analytic method to determine chemical composition, concentration and size of NPs in biological tissues has been developed [61]. Recovery rate of 73–89% were obtained when PS and PMMA nanosphers were added into mollusks tissues. This novel method includes the alkaline digestion of protein prior to the analysis by Pyr-GC-MS technique [61].

Nowadays the QC approach and the use of statistics are often included in the works, even if they are often based on non-univocal criteria. This is due to the fact that there are no standardized procedures and the control criteria of the procedure vary according to the approach that the research group has adopted [8,52,59,62,63].

Most of the studies conducted in the laboratory are aimed at understanding the behavior of NPs and solving the problems listed above. The use of standard polymers is often connected to the construction of databases and fingerprints aimed at the characterization of individual polymers [51,64], or the identification of molecular fragmentation [55], or the products of thermal and thermo-oxidative decomposition [53,59].

### Analytic Instrumentation

Many analytical techniques can be applied to characterize NPs and MPs, some of which are consolidated such as FTIR especially for MPs, while others are still in the experimental phase and therefore there is insufficient information to establish their reliability. The availability of one tool (instrumentation) rather than another, the need to use non-destructive analyses or the uncertainty of an analytical method are critical factors that must be considered from the beginning. In Table 1 some techniques mentioned during the scientific literature overview are reported.

## 3. Real Samples-Sampling

Collecting a sample is the first step in the analytical process and often it is underestimated. A “sample” is constituted by the analyte (compounds of interest) within the matrix generally rich in unwanted substances and which can constitute interferers. Wastewater, drinking water, sediments, soils, sludge, compost and even atmospheric deposition are some examples of different environmental samples.

The choice of the analytical method is strongly influenced by the matrix therefore for the same analyte in different matrices, the method and its operative steps could be completely different.

For the above reason the sampling must be planned and it is necessary to avoid errors because they can affect all the following analytical procedures. The first phase is to frame the problem by marking out the objective, and collect information to define the state-of-the-art. Unfortunately, the research of NPs is still in its infancy and there are no standardized procedures [65]. One attempt of standardization of micro-plastics sampling procedures was design by the National Oceanic and Atmospheric Administration (NOAA), which focuses on water and sediment matrices. Probably this is the reason for which the majority of the literature concern the analysis of micro and nano plastics in marine environments [65]. Unfortunately, due to small particle size of NPs, a lot of actual sampling methods for MPs are not suitable and unemployable for them [35]. Another point to face is the representativeness of the sample; the latter must be homogeneous in composition and able to describe the entire study area. Since MPs and NPs are distributed in the environment matrices in a non-homogeneous condition, the use of appropriate sampling procedures is mandatory to get accurate results [65]. The variability of the sample not only affects its composition, but also their distribution and concentration. Monitoring sites should be randomly selected from appropriate strata and position to provide a statistically relevant dataset. The amount of the analyte inside the matrix define the quantity and the number of the sample to collect [36]. Besseling in his study shows how particle diameter, collision frequencies, attachment efficiency, biofilm, polymer density, burial, and degradation are among the parameters that bring great variability between the samples [66]. The sampling mode will clearly change from matrix to matrix but the ultimate goal will be to obtain a homogeneous and representative sample of the area studied.

To provide a complete work, some parameters of the sampling should be assessed: the position (GPS coordinates), the dimension of the area, the period of the year and the sampling frequency, type of sampling (punctual or average sampling), the number of samples, and the volume so that it is reproducible and reliable, and the spatial coverage can be monitored [36]. The degradation process of MPs into NPs is not clear, but it is certain and unstoppable. The quantity of NPs is destined to increase over time, therefore it is important to study a procedure for monitoring them since there are no actual ones [35].

### 3.1. Water

When it comes to plastic in water it refers to seawater, freshwater, wastewater, and drinking water (both bottled and tap water).

But if for MPs there are standardized methods for the nano fraction an adequate analytical methodology is still lacking. Currently, the commonly accepted methods are those referred to NOAA Technical Memorandum [67]; in Europe, those proposed by the EPFL (Ecole Polytechnique Federale de Lausanne) are also in force [68]. One of the objective of the Life project Blue Lakes (Italy and Germany involved) is to develop a standardized monitoring protocol for the MPs and NPs in the lakes and wastewater treatment plants [69,70].

#### 3.1.1. Surface Water

Almost 80% of plastics in marine environmental are due to land debris [5]. Distribution of micro plastics in marine environment depends on some of their properties, namely size, shape, and density. Different density allows the MPs and NPs to accumulate on the sea/freshwater surface layer, or at the base of the main thermocline. Sea waves are another factor that influences the distribution of micro(nano)plastics along the water column. For this reason it is important to carry out sampling in sea/lake calm conditions [71]. As far as dimensions are concerned, flow models developed for nanoparticles have shown that plastics in the milli-nanometer range are more retained in the sediments of rivers. They tend to aggregate with suspended solids and settle before reaching the sea, limiting their contribution [72].

Contrary to the marine environment, plastics in freshwater systems were poorly studied, until now (see Figure 1 and Supplementary Material Figure S1) although they play a key role for both MPs and NPs diffusion in the environment.

It is possible to find studies where the sampling of surface water is carried out directly with a cleaned HDPE (high-density polyethylene) or glass bottles [73]. Among the most used water sampling techniques are floating trawls: plankton, Neuston, Manta, and Bongo nets [58,74]. They consist in a surface net used to collect suspended plastic debris in water. Unfortunately, their ability to sample NPs is limited by their mesh size between 280 and 450 μm.

In 2017, it has been already highlighted how floating trawls underestimated smaller particles [74]. Besides that, it remains the most used and accepted approach for determining pelagic MPs, zooplankton, and for the comparisons of their abundances [71,75,76]. Floating trawls, are versatile, and can be obtained on different harnesses, although their mesh integrity must be checked frequently.

For river samplings, the trawl is fixed on a bridge after a straight stretch of the river. Samplings are performed in 15 to 30 min; in the meantime, the flow is recorded by a flow meter. Finally, the results are given in particle count and mass per filtered cubic meter and per hour [77]. A particular floating trawl is proposed by Eriksen, M., et al. [78]: AVANI trawl (All-purpose Velocity Accelerated Net Instrument). This kind of trawl is characterized by a good stability at higher speed (up to 8 knots compared whit the traditional one which reach 3–5 knots [68]) therefore can be used in unfavorable atmospheric conditions too. It allows covering larger sampling areas (more than 130 km). Furthermore, its geometry is useful to sampling the water column in addition to the surface. A promising technique, developed by 4H-JENA engineering GmbH, is the direct fractional pressure filtration for large water volumes (>1 m^3^). The cascade filtration allows to perform simultaneous sampling of different size fractions of MPs down to <10 µm [79]. The method applied for MPs could be adapted to NPs.

Another aspect not to be underestimated is the simultaneous sampling of zooplankton and MPs allowing the comparisons of their abundances [75]. In order to have a sample with more representative distribution of plastics abundance on the surface water, a combination of bulk and floating trawls technics (using different mesh nets) is used [80].

The volume of water to be sampled changes according to the size of the studied plastics. Some studies have shown that nano plastics are more abundant due to their size, and smaller water volumes are needed [81]. The study by A, Koelmans et al. [82] recommended to collect volumes depending on the kind of water sampled. Surface water: have a rather low concentration of particles with dimensions >300 μm (10^−3^ to 10 particles per liter). Furthermore, a minimum sampling volume of 500 L is suggested for surface waters that can be increased for lakes and rivers with very low particles concentrations [82].

#### 3.1.2. Wastewater

Considering the anthropogenic water cycle, high concentration of NPs and MPs can be found in municipal sewage and, therefore, in the wastewater treatment plants (WWTPs), practically the link between anthropogenic derived and freshwater systems. The WWTPs are not designed to remove plastic particles and therefore they discharge into rivers and lakes before reaching the sea [66]. Wastewater really is a complex matrix therefore to characterize the NPs and MPs, procedures such as sampling, treatment to extract plastics, and analytic techniques are critical: sampling of influent, effluent, and sludge are included. The wastewater treatment process is a complex sequence of steps. In particular bar screening, degreasing, flotation, biological and chemical oxidation, primary and secondary sedimentation are included. NPs and MPs can be partially removed by trapping in sludge sedimentation or during the degreasing processes therefore their removal efficiency can be extremely variable. Sampling can be performed utilizing mesh screens (from 25 to 500 μm) shacked on top of each other [83]. A continuous sampling device to collect wastewater has been developed by Dyachenko et al. and by Talvite et al. [84,85]. Since wastewater usually contains a high number of particles, a volume of 1 L is sufficient and it is not recommended to exceed 500 L for effluent water to avoid clogging of the sieve during the filtration [82].

Sludge generally contain solid materials and high content of NPs and MPs, and can be sampled by a Van Veen grab sampler usually used for sediment sampler. Degradation of the organic matter represents the limiting step for the NMPs extraction from sludge. To separate NPs and MPs from the solid matrix sludge can be washed by water through a series of mesh screens [86]. Recently it has been observed that the extraction of MPs from a sludge can be lower than other solid matrices, a chemical oxidation by Fenton reagent, HNO3 or HCl can destroy the flocs improving the NMPs extraction [87].

#### 3.1.3. Drinking Water

MPs have recently been found in the drinking water, PE = PP > PS > PVC > PET polymers were found in drinking water samples [82].

Unlike surface water, drinking water samples are collected manually or pumped into glass bottles, but automatic composite samplers are also available. All the collected water samples are filtered in laboratory and the filters are analyzed [82].

Considering tap water, the sampling volumes change, a minimum sampling volume of 1000 L is recommended (range between 10^−4^ to 100 particles per liter) [88]. While for bottled water containing very small particles (<100 μm) 1 L is enough and for the larger particles more than 10 L are required (normally multiple bottles are analyzed merging them as single sample) [82].

### 3.2. Soil

Activities related to the agricultural in farms appear as a relevant source of micro(nano)plastic in soil, especially related to the plastic mulching or sewage sludge. According to the 86/278/CEE EU directive [89] the use in agriculture of sludge from wastewater treatment plants has been encouraged. This last is the outcome of industrial residual semi solid material and municipals wastewater after of sewage treatment. More than 50% of sewage sludge is reused as a fertilizer, which is combined with natural soil, so micro(nano)plastic components are dispersed across a wide spatial area. Just in 2016, it has been estimated that the production of micro(nano)plastic was between 63,000 and 430,000 Tons only in European farm lands, equal to 125–850 tons of microplastic per million inhabitants added [90]. In addition to this, some polymers such as expanded polystyrene foam (EPS) and polyurethane are used as additives to improve soil properties in particular in the horticultural sector and foam does not break down easily in the environment. These ranges (63,000–430,000 tons and 125–850 Tons) are so wide because they include city debris degradation and tire wear fragments. Micro(nano)plastics may be produced in situ through the breakdown of larger plastic debris or transported by atmospheric agents to long-range distances [91]. Fluvial, lake, sea sediments and shoreline have been shown to contain concentration of micro(nano)plastics [36,80,92,93]. The monitoring of marine, lake and river environments often include sediments analysis. This method is certainly cheaper and simpler to act than sea water sampling [36]. The degradation mechanisms of plastics in soil are still under study and no standardized procedure has yet been developed.

According to literature, first of all, to collect samples the studied area is divided into squares at regular intervals, which occupy the entire length. Sampling along coastal area can be excluded for its length; Lippiatt et al. required a minimum of 100 m in length parallel to the water [36]. Alternatively, it is possible to adopt different methods based on the length of the coast as for Mattsson et al. who analyzed in the same work different length of shoreline. The sample (square of 0.3 × 0.3 m) is divided into four parts if the coast is less than 100 m length, otherwise the samples (0.3 × 0.3 m) are collected every 15 m [19]. After dividing and defining the geographical target area (including the GPS coordinates), a distinction must be made between superficial evaluation of a single deposition event or in-depth sampling to study the stratification over time. Normally superficial sampling includes depths between 5 and 10 cm performed with steel shovel [68,94,95]. Otherwise, deep sampling can be performed according to a core drill procedure. Ceccarini et al. collected core of sand by using cylindrical glass vessels of 17 cm height and 11 cm of diameter while a stainless-steel vessel (h = 25 cm, diameter = 8 cm) were used to take sand samples along the Castelporziano beach (Rome Italy) [96].

Until now the surface area was the only parameter considered, but it is also possible collecting different samples with the same weight. This new parameter is not fixed, but variable with the laboratory method: Mintenig et al. collected 0.5 kg of sewage sludge using steel shovel [97], on the other hand, Castelvetro et al. sampled 1 kg of sediment from shoreline lake [95] and Ceccarini et al. picked up different carrots of 0.5 kg from the Venice lagoon area [77].

Mauricio Díaz-Jaramillo et al. [54] conducted a study to evaluate the abundance of MPs in deeper sediments from SW Atlantic Argentinean estuaries. Sediment samples were collected by an aluminum tube core of 100 mm diameter and 50 cm length. The core samples were divided according to three depths: surface 0–10 cm, medium 10–20 cm, and deep 20–30 cm. Sediment samples were immediately covered by aluminum foil, and frozen at 20 °C. MPs were extracted from sediment using the sediment microplastic isolation unit (SMI) described by Coppock et al. (2017) and using a zinc chloride (ZnCl2) solution and later to visual classification and photography, the polymer composition was analyzed by attenuated total reflectance (ATR) Fourier transformed infrared spectrometry (FT-IR) [57].

Standing-stock and random surveys samplings methods, included in the NOAA MDP and UNEP/IOC (United Nations Environment Program/Intergovernmental Oceanographic Commission) guidelines are compared by Lippiatt et al. [36]. Standing-stock surveys quantify the debris over time, each survey event records the debris in a specific moment. Debris is removed from the sampling area at the start of monitoring and they cannot be removed until the sampling event. Lippiatt et al. for the shoreline standing-stock surveys suggest once every 28 ± 3 days [36]. Random samples can be collected from sandy beach locations in 1 m^2^ for analysis of meso- and micro- debris. Since meso- and micro-debris concentrations are very patchy, random samplings may not always be the preferred method. The situation for NPs is even worse, not only because of irregular distribution but because the exact interactions are unknown [36].

Sand and agricultural soil samples were analyzed using pyrolysis–gas chromatography/mass spectrometry (py-GC–MS) respectively. Jia Lin et al. [59] have conducted a case study for quantifying MPs in marine sediment showing the historical variation of MPs in the sediment core (1925–2009). The sediment samples were initially dried, sieved, and divided into two size fractions, i.e., >1 mm and <1 mm. MPs with a size of >1 mm were sorted manually, while the other fraction was processed via organic matter digestion, MP extraction, and acidification for purifying and concentrating the MPs before measuring the microplastic derived carbon (MPC) contents. The abundance of MPs for each sample was expressed by the MPC content measured by a carbon-sulfur analyzer.

Davranche M. et al. [46] demonstrated the presence of NPs in sand water extracts (SWEs) and the relevance of developing geochemical tracers for determining the fate of missing plastic litter. In this case, plastics were collected directly from the beach using a net of 100 nm mesh (o mesh o nm). After fusion of the sample with LiBO_2_ and acid digestion with HNO_3_ the analysis was performed by inductively coupled plasma-optical emission spectrometry (ICP-OES) and by inductively coupled plasma-mass spectrometry (ICP-MS). Furthermore, in this innovative work the authors demonstrated that NPs are important components in the rare earth elements (REE) signature of coastal sand (29 to 73%). In fact, Europium (Eu) can be used as a marker as it is part of the additives used during industrial processes. A geochemical approach is therefore useful for studying the trace of plastic debris in the environment [46].

### 3.3. Atmosphere

From 2019 to 2021, several works dealt with the analysis of MPs and NPs in the atmosphere. Given their persistence and low-density characteristics, NPs are subject to long-distance transport worldwide, even away from the primary source. This is supported by observations of MPs in remote (Arctic, Swiss Alps) and urban (Bremen, Germany) snow, although estimated annual deposition in these regions was low (average 1.4–66 MPs/m^2^) [60,98].

Compared to MPs, nanoplastics have very different characteristics that also influence long-distance transport and migration, which is accentuated [99].

Atmospheric deposition can be distinguished in wet (rain and snow) and dry (particulate matter-PM), and both scavenge the atmosphere [100]. The analysis of NPs in the atmosphere is not limited to the sampling of air, but it includes the depositions too [49,51,63,101].

MPs in the air are assumed to pose a health risk: small plastic particles and fibers could be breathed in and may settle in the lungs of animals and humans [63].

The even smaller size of the NPs favors their penetration and, entering the capillary blood system, they are distributed throughout the body [99,102].

Some studies show the danger of NPs produced by the masks used in this period of COVID-19 [103]. It has been found that POPs and other harmful compounds accumulate on MPs, such as trace metals and pathogens [104,105]. It is suspected that NPs may also be a vector for the coronavirus [99].

Owing to their nanometric size and fibrous shape, plastics can reach the deepest respiratory tract. The Terzano et al. study proved the toxicology risk during inspiration, inoculating micro(nano)plastics polymers in lung tissues [103,104]. Obviously, the smaller the size of the polymer particles in indoor air, the harder it is to isolate them.

Active and passive, indoor and outdoor atmospheric sampling are included and for both, given the small size of NPs, a cascade sampler that divides gradually decreasing particulate sizes into several plates has been hypothesized [62].

#### 3.3.1. Outdoor

A pump sucking the air at a defined flow performs active sampling, normally there is a cutting head (PM_10_ or PM_2.5_) where the particulates with aerodynamic diameters lower than the cut-point are selected and the filter (where the sample is collected) is then analyzed in the laboratory. For sampling the most used sampling media are: quartz fiber filters (pore size: 1.6 µm), glass microfiber filters (pore size: 1.6 µm), or polytetrafluoroethylene (PTFE) filters (pore size: 2 µm) [60]. For air sampling a volume range between 2 and 23.93 m^3^ has been reported [60]. If head for cutting particulate matter is not used and total suspended particulate matter is collected, a treatment will be necessary to separate the NPs from those of higher cutting [101].

Passive sampling includes snow/rain sampler and particulate fallout collector or ambient filter sampler. These instruments are placed on the ground or in the air over a period of time. The height at which the collector is fixed, the geographic coordinates of the collection, and the volume of air sampled must be considered [60,65]. Passive samplers for rain sampling basically consist of a bottle and stainless-steel funnel. After collecting the sample, the funnel is often rinsed with deionized water to remove all particles adhering to its surface [106]. An example is the research performed in London to monitoring MPs in air, which was settled collecting twice a week for 4 weeks depositions. An aluminum rain gauge (200 mm diameter) was used which was continuously exposed, resulting in three or four-day sampling periods. Data regarding temperature, relative humidity, wind speed, and direction were collected. Once the exposure time was over, the samples were transported to the laboratory and stored at 4 °C. The treatment was carried out for maximum 4 days after the sampling [107]. Some seasonal studies have involved the city of Paris, determining considerable concentrations: from 29 to 280 particles/m^2^/day [108]. The study carried out in Hamburg (Germany) on the deposition of MPs also included a long period (from December 2017 to February 2018). For the collection of the samples, nine mass samplers were used. The bulk samplers were installed 100 cm above the ground level at least 100 cm apart. Again the samples were collected with passive PVC samplers for rain (funnel and bottle) [109]. The data reported by the city studies are quite similar to each other, but the absence of this kind of study does not allow to understand the transport of NPs at a global level [65].

Matrices closely related to the atmospheric behavior are snow and ice. These can provide temporal and seasonal information on global transport and information on the fractional deposition (altitude and latitude), especially in relation to remote areas such as the mountains, the Arctic and Antarctica [51].

Ice samples are collected with core drills or stainless-steel cylinders. The modalities are not unique but often include core drilling at different depths as for Costa et al. (2.6, 2.8, and 3.0 m) to avoid external contamination [51]. In addition, 1 cm outside of the surface is shaved with a clean ceramic knife. To avoid possible physical degradation of the polymers, samples were filtered with a PTFE filter with pores of 0.2 μm to separate the MPs from the NPs [51]. The laboratory blanks consisting of Milli-Q exposed during the sample handling were carried out.

About snow, the surface collection technique can also be added if a single deposition event is enough for seasonal sampling. Snow collection is arranged by defining the sampling areas and the sample once collected is melted and treated as freshwater.

The quantities of samples to be collected has been higher than those taken for freshwater to detect the chemical pollutants [51,110]. To reveal PET in the Alpine area the collected sample undergoes membrane filtration followed by thermal desorption-proton-mass transfer [49]. In this case study, a polypropylene container was used to collect the snow. To distinguish between wet and dry deposition, Materic et al. sampled the snow surface immediately after a snowfall and the same surface was collected after two days in the absence of precipitation. The difference between the two samples was attributed to the contribution of dry deposition [49].

#### 3.3.2. Indoor

For indoor air, a sampling pump can be used for sample collection. An example of such pump is the stand-alone sampling pump used by Dris et al. [91]. Whereas, for deposited MPs indoor, dust can be sampled by a vacuum pump or vacuum cleaner normally used in homes and dust particles collected in the cleaner bags [106]. To collect MPs in dry atmospheric fallout (dust fall) in indoor environments, quartz fibers or double-sided adhesive plastic pads have been used as passive samplers. Dust samples were collected using vacuum cleaner, hog bristle brush, or wooden brush, and then transferred to low-density polyethylene bags or paper bags. To avoid cross contamination, the brushes used to collect dust should be washed with filtered distilled water or rinsed with ethanol [60]. Given the time we spend indoors (workplace, entertainment places, home), studies in this area are multiplying. Many of which concern not only cosmetics and cleaning products but also fibers from clothing [63]. O’Brien et al. using 55 m^3^/h active samplers on a glass filter (1.6 μm), fired in a muffle at 450 °C for 4 h before use, he studied the emissions of a washing machine and dryer. Despite the novelty in the type of study, the sampling and the analytical method follow a QC through field and laboratory blanks [63].

Description of samples and analysis related to the atmosphere compartment is reported in a review by Enyoh et al. [106].

### 3.4. Biological

Following the NPs diffusion in the environment, all animal species can be involved in a direct way by ingestion and through the bio-magnification [19]. The plant kingdom is also included within the study because it is closely related to soil pollution [111,112] therefore sampling biological matrices to understand dose-effects of NPs is fundamental for wildlife safeguard.

Particularly noteworthy, although not directly sampling linked, are laboratory studies. It is possible inoculating standard polymers in shellfish, fish, algae, or plants coming from aquaculture production to study laboratory processing techniques, trophic processes, and interaction of micro(nano)plastic with cellular tissue [113,114]. Long et al. in their study involved three species of algae: *Chaetoceros neogracile* (5.3 μm) and *Tisochrysis lutea* (4.5 μm) obtained from the Scottish Marine Institute and *dinoflagellate Heterocapsa triquetra* (18 µm) was isolated in the Penzé River. All species were exposed in glass flasks to pristine 2-µm polystyrene beads (yellow-green fluorescent, density of 1.05 g mL^−1^) uncharged and smooth to two deionized water solutions at different concentrations (3.96 µg L^−1^ and 39.6 µg L^−1^). Prior to sampling the mix of algae and polystyrene was homogenized every day though stirring [115].

As much as concerning plants, nanoparticle have a size compatible with the plant cell wall pores, therefore they can migrate by osmosis or capillary action [116]. They can also interact with membrane proteins and transport of ions. The main ways through which NPs and plant can interact are: cell wall and cuticle by passive transport; proteins on the membrane by diffusion facilitated; endocytosis; plasmodesmata by intercellular translocation; stomata [111]. Laboratory test exposure of plants to NPs has basically the same procedure as animals. A dispersion of the standard polymer is prepared in deionized water and the organisms are exposed according to different times, concentrations, and additives depending on the laboratory procedure. Lin et al. prepared a 5% (*w/v*) dispersion of fluorescent (Nile red) and non-fluorescent nanospheres (100 nm), in deionized water. Wheat seeds (*Triticum aestivum* L.) were soaked in non-fluorescent polystyrene nanoplastics (PSNPs) dispersions (0.01, 0.1, 1.0, 10 mg/L) for about 2 h at room temperatures. Prior to exposure the seeds were sterilized by 2% (*v/v*) hydrogen peroxide solution for 30 min and rinsed with deionized water. Subsequently, 25 seeds were incubated onto a Petri dish for 5 days, in a growth chamber under dark condition at 25 °C. During the incubation period the germination of wheat seeds was recorded. First evaluation was the sprout and root length of wheat seedlings [112]. Laboratory tests of incubation and inoculation are fundamental to establish the correct procedure to apply to real samples and minimize errors.

A method used for quantifying micro(nano)plastics in marine environmental samples is zooplankton and phytoplankton sampling analysis. Plankton samplings offer the possibility of studying both benthonic and nencthonic behavior and to defining trophic transfer from prey to predator [117,118]. Plankton is normally sampled through fine mesh nets. This one can be used to sampling directly NPs too. Plankton net trawls capture particles in range of tens to hundreds of micrometers. It means that concentration of NPs is underestimated in aquatic compartment. According to the literature, since nanofibers are not retained by the most used plankton net, they are partially included in NPs count, despite fiber are prevalent than particulate [114]. According to Abdolahpur Monikh et al. [119] micro(nano)plastics and POPs tend to accumulate in lipid tissues. For this reason, eggs shells represent an excellent way to monitor the bio-accumulation in females of a species [119]. Post-mortem examinations are among the methodologies applied above all for higher-order animal organisms. This procedure can be performed on site if it deals with large mammals (generally, necropsies are conducted on stranded cetaceans) or in the laboratory by taking samples. Unfortunately, the lack of a real protocol leads to the loss of the smallest particles. When studying gastro-intestinal tracts (GITs), usually the lower size limit is 1 mm as reported for whales and dolphins [120]. Getting to a standardized procedure would allow comparisons between the results of different studies of mammals, birds, and fish [120]. The work done in Ireland reports an analysis over a time period from 2015 to 2018 in three main compartments of the digestive tract: esophagus, stomach (including forestomach, fundic stomach, pyloric chamber, and duodenal ampulla), and intestines. From time to time the procedure has been refined recording the number, color, size, and shape of the micro and nano plastics found. Through these studies, sieves for micro plastics up to 200 mm have been introduced [121,122]. Direct observation for morphology diagnosis are performed by stereomicroscopy that is applied in both animal tissue as mussels [123] and vegetable, one as surface of seeds and germinated roots [112]. Alternatively, the samples of tissue are processed in laboratory through acid attack and/or enzymatic digestion [47,124]. Small mussels, zooplankton, and plants are totally processed while tissue samples are usually collected from bigger mammal and birds. Normally animals tissue samples are collected from the gastrointestinal tract and from the liver, organs in which there is the greatest accumulation of micro (nano) plastics [10,47,123,124]. Finally, organisms at a higher trophic level belonging to the same habitat such as fish and birds can be considered together. To describe Lake Geneva, Faure et al., sampled 40 fish and nine birds (three swans, one grey heron, and five mallards) swans to analyze their stomach tract by a stereomicroscope [68]. Having a single standardized procedure would allow not only the application between different species, but also the implementation of a research with the data of other studies.

## 4. Pretreatments

The pretreatment of the sample is a necessary step because it often helps to reduce the interference due to the matrix and can be aimed at separating the NPs and MPs by classes of polymer. In complex environmental samples, natural organic materials and non-plastic particles can form homo or hetero-aggregates with micro and nanoplastic therefore it will be necessary to remove the matrix. Furthermore, the density of organic matter in soil is usually similar to that of different plastics (e.g., PET and Nylon), which is why simple density fractionation is often not sufficient [125]. Regardless of the technique used, it is possible to define three main groups: digestion, preconcentration, and separation [3,12].

Anyway, all treatments must respect certain points, i.e., not alter the characteristics of the plastic materials such as weight, number, or shape [79,126].

NOAA or EFPL suggests the following sequential steps for the analysis of some MP polymers (PE, PP, PVC, and PS) in water-sediment samples [67,68]. All the treatments that the sample can undergo once in the laboratory are listed below.

### 4.1. Digestion of Matrix

Degradation and digestion of organic matter are the most used techniques to extract micro and NPs from complex matrices such as food, biological sample, soil, and sediment. All the treatments to which plastic samples are subjected must not alter their characteristics: weight, number, or shape [126]. To recover MPs from biological tissues, digestions using acid-base, oxidant, or a mixture of both can be used successfully. Several digestion protocols were explored such as 65% nitric acid [127] sometimes in combination with 30% hydrogen peroxide and sodium hydroxide [12] or potassium hydroxide (KOH) for the alkaline treatment [64,125]. Few publications only mention the use of other strong acids like hydrochloric acid [128]; the acid treatment, has the advantage to destroy also some inorganic particles like carbonate (especially for the sediments) in addition to the organic matter [12]. In the milder treatments, hydrogen peroxide is used as oxidizing agent, using the same methodology proposed by Ecole Polytechnique Federale de Lausanne (EPFL) or NOAA guide (Wet peroxide oxidation) [67,68]. It is possible to combine heat treatments to improve digestion. Defu He et al. [129] published a comparative study between 10 M NaOH, 10% KOH, 30% H_2_O_2_ solutions and the Fenton’s reagent for the removal of organic material from soil to analyze MPs. The use of this reagent combined with the separation by density has shown excellent results. The Fenton reagent was used with enzymatic digestion for the treatment of waste water samples by Nguyen and co-authors [125]. Several plastic polymers (e.g., polyamide, polyoxymethylene, polycarbonate) can be degraded or damaged by these acid/base treatments (particularly at high temperature). A novel thermos analytical method has been used to remove organic matter followed by an acidification procedure to enhance the purification of MPC by removing inorganic carbon (e.g., carbonate minerals and shells) with >85% phosphoric acid [59]. Acid, alkaline, and heat treatment could provoke aggregation of the particles, most likely due to the strong change in ionic strength of the solution [126,127]. A recent study confirmed that the majority of MPs were unaffected by the H_2_O_2_ digestion at 70 °C [92], while the morphology of some microparticles, such as ABS, PA, and PET, was changed by action of HNO_3_ [69,130], which limits the applicability of these reagents. Correia M., et al. [47] investigated NPs in fish samples testing two sample preparation strategies: acid digestion and enzymatic digestion with proteinase K. In this study they showed that acid digestion causes aggregates/agglomerates (>1 μm) unlike enzymatic treatment.

To avoid degradation/aggregation processes, sequential enzymatic digestion was tested for plastic samples purification. Enzymatic separation could also be a good option for NPs because it does not alter the plastic particles and stabilizes them against aggregation. Cole et al. [128] compared treatment with HCl, NaOH, and enzymes (proteinase- k) in zooplankton proving that the enzymatic treatment degraded about 97% of the biogenic matter and at the same time did not change the size of the microplastic. In another study [79] the sample purification with different enzymes (lipase, amylase, proteinase, chitinase, cellulase) have been successfully tested for subsequent micro-FTIR spectroscopy.

The air samples mainly consist in filters, for active sampling, or in depositions, for passive samples. To treat the dry deposition samples, the collection equipment (funnel and bottle) is washed with deionized water and eventually solvents to remove particles from the surface of the equipment. The procedures provide the vacuum filtration using silver membrane or cellulose filters and left to dry for 24 h at room temperature [79] (or at 40 °C for approximately 4 h [107]) in glass Petri dishes. The same pre-treatment can be done with wet deposition (water and snow) and ice cores [110]. In order to effectively stain the particles, the Nile Red staining protocol established by Tamminga et al. [131] was applied. The binding of this lipophilic dye to the hydrophobic surface of plastics allows a more targeted fluorescence analysis of the sample, for these samples, NaClO was used to destroy the organic matter.

### 4.2. Preconcentration

To improve the limit of detection (LOD) and limit of quantification (LOQ) of existing methods, sample preconcentration can be applied [132]. A preconcentration step is inevitable because NPs are tiny, low-weight particles. Depending on the type of sample and identification, it is possible to choose between different types of preconcentration. Specific techniques for the analysis of nanoparticles are: membrane filtration, ultrafiltration, ultracentrifugation, continuous flow centrifugation (CFC), cloud point extraction (CPE), and evaporation of solvent [12,41].

### 4.3. Filtration

Among the drawbacks that this technique certainly presents there are problems of interference and contamination due to the same polymeric material used for filtering. The volume to be sampled is inversely proportional to the size of the pores, therefore it is not a particularly suitable technique in the case of environmental samples rich in organic matter [12]. Many studies suggest cascading sieves of different mesh sizes to separate size classes and facilitate the quantification of MPs [111,112]. In these cases, cascade filtration with membranes of different pore sizes or ultrafiltration is much more suitable. Air samples (both passive and active) as well as precipitation (snow or rain) or ice cores are filtered and subsequently the filtrate was collected [110]. Silver membranes or cellulose filters vacuum pumps are used and dried for 24 h at room temperature [79].

### 4.4. Ultrafiltration (UF)

These techniques are indicated for large sample volumes and consist in the use of nano-porous membranes that have a molecular weight cut-off in the range of 10e100 kDa (5e50 nm). An important advantage of this method is that the particle loss, sample alteration, or aggregation are minimized because in this technique the solvent is not completely eliminated [41].

### 4.5. Centrifugation & Ultracentrifugation (UC)

It is not particularly suitable for environmental samples as it is limited only to small sample volumes of the order of 10–100 mL.

Hildebrandt et al. have proposed a study in which they used continuous flow centrifugation (CFC) to sample MPs in natural waters with excellent results on at least 6 types of polymers [133] demonstrating its suitability for volume-reduced MP sampling. Another study successfully proposed two CFC sequentially at different rotational speeds for the preconcentration of commercial Pd-doped PS nanoplastics in river water samples [73].

For biological samples, generally, ethanol is used to separate plastic from less dense matrix but ethanol can dissolve or damage some types of polymers, especially smaller particles. A good alternative is density separation by centrifugation, for example using suspended colloidal silica nanoparticles (Percoll) as a density gradient medium [125].

### 4.6. Could Point Extraction (CPE)

The CPE employs a surfactant solution to separate and preconcentrate inorganic nanoparticles, particularly for the nanoparticles with hydrophobic coatings. This technique does not change the original size and morphology of nanoparticles in a complex matrix and is a simple, safe, inexpensive, and nonpolluting approach. Xiao-xia Zhou et al. in a study proposed the CPE method based on surfactant (Triton X-45) to preconcentrate trace NPs in environmental waters [132].

### 4.7. Evaporation of Solvent

Often this step is necessary to pre-concentrate suspensions of NPs, with a rotary evaporator. It is not an economical technique especially in the case of large volumes but can be used in combination with other preconcentration techniques such as dialysis or cross-flow UF [12].

### 4.8. Density Separation

Plastics can be separated from the soil matrix through salt solutions. Soil samples are mixed with high-density solution, centrifuged and let it to settle until plastic particles float on the surface and the denser soil materials remain on the bottom. Plastics floatation can be achieved passively or assisted by elutriation [134] or ultrasonic treatment [135]. The saline solution should be chosen according to a compromise between particle recovery, processing cost, and environmental impact. In a recent study, Zhang et al. [136] developed a simple and cost-effective extraction method from soil samples suitable for light density plastics (PE and PP). According to Liu et al. [67] the NaCl solution (density, 1.18 g/cm^3^) is an effective way to float out of the matrix MPs, but it is not suitable for high-density plastics such as PET or PVC. It is suggested that the optimal density values of a solution should be between 1.6 and 1.8 g/cm^3^, achievable with ZnCl_2_ or NaI [134], although more expensive than other salts. Likewise Defu He et al. [129] proposed an alternative extraction to that with NaCl, using CaCl_2_ obtaining a relatively high efficiency. Normally, the improvement of NaCl extraction methods is preceded by ultrasound treatments to extend the floating time [135]. Claessens et al. [134] have developed a new technique based on the principle of elutriation, i.e., a process that separates the lightest particles from the heaviest ones through an upward flow of gas or liquid. After this, the sample is extracted with NaI, stirred and centrifuged. The authors suggest repeating the extraction process two to three times to obtain an optimal result. As an alternative to flotation by density through saline solutions, the oleophilic properties of polymer can be exploited through oil extraction. This method has been reported by Crichton et al. for the extraction of MPs from sediments [137]. The addition of Nile red dye increases the detection of plastics by microscope determination [5]. The sediment-microplastic isolation (SMI) unit has been proposed by Coppock et al. [57] to separate MPs from sediments of differing types, using the principle of density floatation. Zinc chloride is suggested like the most appropriate floating agent because it is cheap and at the same time allows the sedimentation of the finer particles and the flotation of the denser ones. The method was validated by artificially spiking sediment with low and high-density MPs, and tested by extracting plastics present in natural different sediment samples (fine silt/clay and coarse sand). The SMI is cheap, reproducible, and is easily portable because it is characterized by a small dimensions and low weight. These characteristics make it extremely versatile and suitable for both laboratory and field instrument such as onboard research vessels.

### 4.9. Field Flow Fractionation (FFF)

FFF is a separation technique typically for size and molar mass fractionation of biopolymers, proteins, polymers, and nanoparticles. In particular an external field force such as electrical, centrifugal, gravitational, and magnetic is applied perpendicularly to the fluid suspension pumped through a long channel. The separation of particles depends on the different interaction with the property of the sample and in particular the mobility under the external field-induced force. However, the most used FFF for NPs separation in water samples is the asymmetrical flow field-flow fractionation (AF4) where the external field is the cross flow created by the asymmetrical wall. There are various studies that propose the use of (AF4) for the analysis of NPs in different samples. Correia et al., presented a research study of NPs in a food matrix employing AF4 coupled with multi-angle light scattering to detect spiked PS nanoplastics [47]. AF4 can separate and characterize nanoparticles simultaneously through coupling to online detectors, it adapts to various detection methods, can reduce the complexity of samples, and provide information about particles of different sizes [12].

### 4.10. Chromatographic Techniques

All those that exploit the affinity for a stationary phase for the separation of analytes fall under “chromatographic techniques”. Among these techniques one of the most common is the size-exclusion chromatography (SEC) in which NPs are separated according to the relationship between the size of the gel and the size of the NPs. Due to the possible interactions with the stationary phase and the size of the pores, this technique is not particularly suitable for particulate samples because a pretreatment step is necessary to prevent pore clogging [12]. A passive separation technique is hydrodynamic chromatography (HDC) that utilizes hydrodynamic and surface forces to separate NPs in liquid [138]. The important advantage of this technique is the rapid separation of particles only based on size differences, analytical repeatability, and easy operation.

### 4.11. Electrophoresis

The electrophoresis employs the mobility of charged particles in an electric field to achieve a spatial separation. Recently, S. Felsing, et al. proposed an extraction method based on electrostatic properties of plastics for separation from different environmental sample such as water, sediments, and bleach sands [139]. Although the reported studies focus on MPs, these techniques can also be adapted to NPs [19], even better if preceded by a preconcentration [12].

### 4.12. Optical Tweezers

This is a promising separation method to trap NPs for subsequent identification with a Raman microscope. With the tiny forces that light exerts on the matter, the micro and nanoparticles dispersed in liquid can trap and manipulate. The Raman Tweezers (RTs), optical tweezers combined with Raman spectroscopy, as an analytical tool for the study of micro- and NPs in seawater was applied by Gillibert et al. [140]. Specifically, this application concerned the determination of PS, PE, and PMMA particles in size of tens of microns to 90 nm.

The main advantages of this technique are high separation resolution, non-contact with particles, and coupling with Raman spectroscope; however, it still finds limited applications because it can be used for transparent particles only [140].

## 5. Work in Progress–Laboratory Tests, Sampling and Pretreatments

A novel quantification procedure of poly(ethylene terephthalate) PET and polyamide (mainly nylon 6 and nylon 6,6) from wastewater treatment plant sludge was recently described [52]. This innovative protocol consists of depolymerization by acid/alkaline hydrolysis and the quantification is performed by HPLC with fluorescence detection after derivatization of the monomers. The proposed analysis protocol consists of a first Soxhlet extraction of the wet sample with methanol. This step is followed by a further extraction of the solid residue with n-hexane, centrifugation, and drying. Part of the dry sample is then extracted with dichloromethane and subjected to depolymerization. For the acid treatment (nylon 6 and nylon 6,6) after addition of 40 mL 6 N HCl the stirred mixture was heated to the reflux temperature of about 105 °C for 24 h. For PET depolymerization, an alkaline hydrolysis is required, so after addition of 40 mL of aqueous 1.9 N NaOH and TBHDPB as a phase transfer catalysis the mixture was heated under stirring at 85 °C for 6 h. This is an innovative procedure because this is the first approach to applying a method allowing quantification of the total mass of poly (ethylene terephthalate) PET and polyamide (mainly nylon 6 and nylon 6,6) in complex matrices with high selectivity, accuracy, and sensitivity as compared to the conventional quantification by mechanical separation by flotation and identification by micro-spectroscopies. The same authors have used a similar approach for the determination of PET in sediments. The procedure involves aqueous alkaline PET depolymerization with phase transfer catalysis, oxidation, and fractionations to remove interfering species and pre-concentrate the terephthalic acid (TPA) monomer with consequent quantification by reversed-phase HPLC [95].

Duemichen E. et al. recently proposed a fully automated TED-GC–MS system for analysis of gaseous degradation products and thermal decomposition processes of polymers [53]. This is a two-step method in which a sample is first decomposed in a thermogravimetric analyzer (TGA) and the gaseous decomposition products are then trapped on a solid-phase adsorber. In the second step, the solid-phase adsorber is analyzed with thermal desorption gas chromatography mass spectrometry (TDU-GC–MS). In this work different materials were tested such as a sample of low-density polyethylene (LDPE), polypropylene (PP), wood plastic, composite polystyrene, and a PE/PP blend was analyzed as an unknown PE/PP blend and suspended particulate matter (SPM) (provided by the Environmental Specimen Bank of the German Environmental Agency). The experiments proposed in this study demonstrated the potential of this automated system as a routine method for identifying and quantifying MPs with high precision in the environmental samples and in complex matrices such as a wood plastic composite.

Duemichen E. et al. also demonstrated that with the TED-GC-MS it is possible to identify unknown polymers even as a physical blend and to easily quantify their weight ratio by comparing them with reference blends [53].

An evolution of the proton transfer reaction—mass spectrometry (PTR-MS) generally used for qualitative and quantitative analysis of volatile and semi-volatile organic vapors was tested recently [132]. Materić D. et al. [51] presented a highly sensitive method for detection, speciation, and quantification of MPs and NPs polymers based on thermal desorption followed by proton transfer reaction-mass spectrometry (TD-PTR-MS) developing a fingerprint algorithm for polymer identification when present in a complex organic matrix. The samples were loaded in a chromatographic vial previously baked at 250 °C and the low-pressure evaporation/sublimation (LPE) was applied. In order to reduce contamination by indoor environment nitrogen was added. The vials containing the dehydrated samples were loaded into a thermal desorption (TD) unit to enable transfer into the PTR-MS instrument. In Table 1, a list of the techniques utilized for the NMPs analysis has been reported, some of them are still in progress.

For the detailed methodological protocols see the reference publications [49,51,132]. This technique applied for the determination of NPs (e.g., polystyrene, polyethylene terephthalate (PET), polyvinyl chloride, and polypropylene carbonate) in snow samples shows 100 times greater sensitivity than traditional methods, the detection limit of the polystyrene (PS) obtained is <1 ng of the compound present in a sample. All this makes TD-PTR-MS particularly suitable for small sample volumes with the further advantage of to carrying out experiments without a preconcentration step [49].

## 6. Conclusions

The main characteristics and cost-effectiveness of plastics make them irreplaceable materials in our daily life. This paper summarizes the common sampling and handling methods used for the monitoring studies. From this review it can be deduced that in monitoring studies harmonization of sampling and analytic methods are required to allow an acceptable comparison between data regarding MPs and NPs pollution even in different environmental compartments.

In the figure, all the main steps have been outlined in order to carry out an analysis in the most correct way.

The first step should be to carry out experimental designs for the sampling and analytical method, even before getting to the real samples.

For the validation of an analytical method before being applied in the field laboratory tests are conducted.

There are therefore some points that must be respected and include the robustness of the method, the linearity, the precision and accuracy, the selectivity and uncertainty before evaluating the matrix effect factors completely absent in the various works. To get to validate a method, the first step is to use standardized reference materials that also give the possibility to replicate the same procedure in other laboratories. This last factor is completely lacking in the field of NPs. Unfortunately, having standards of NPs or MIX of NPs that simulates those of real samples is completely impossible since polymers are subject to variables such as the state of aggregation, the interactions between them, degradation by UV radiation, mechanical or bacterial degradation. This also changes according to the type of environment (sea, river, lake, soil, altitude, latitude, temperatures, etc.,). Furthermore, NPs can adsorb on the surface and be a carrier of both pollutants and bacteria (for which they offer an excellent nutritional surface) [99].

This review found that in other papers researchers faced great difficulty in quantification not only in terms of identification but also accuracy and precision. The absence of certified reference standards does not allow any of the methods indicated to affirm the goodness of the accuracy of the method used, while the precision is often undermined by contamination during the various sampling and pretreatment phases. It is in fact essential to check during these phases that the materials of all the instruments, the clothing of the operators, the environment in which the sample is processed, and the state of conservation of the sample do not interfere with the analytical target.

The mesh of the analytical target represents a further obstacle, currently, the methods of the MPs are adapted to the NPs. Unfortunately, as seen in the literature, the dimensions of the NPs influence their characteristics, distinguishing them from the MPs by the state of aggregation or migration between the different environmental compartments.

On the basis of this review work, it is considered more appropriate for the analysis of nanoplastics to follow the approach used for engineered nanomaterials rather than that for microplastics [34].

**Figure 3:**
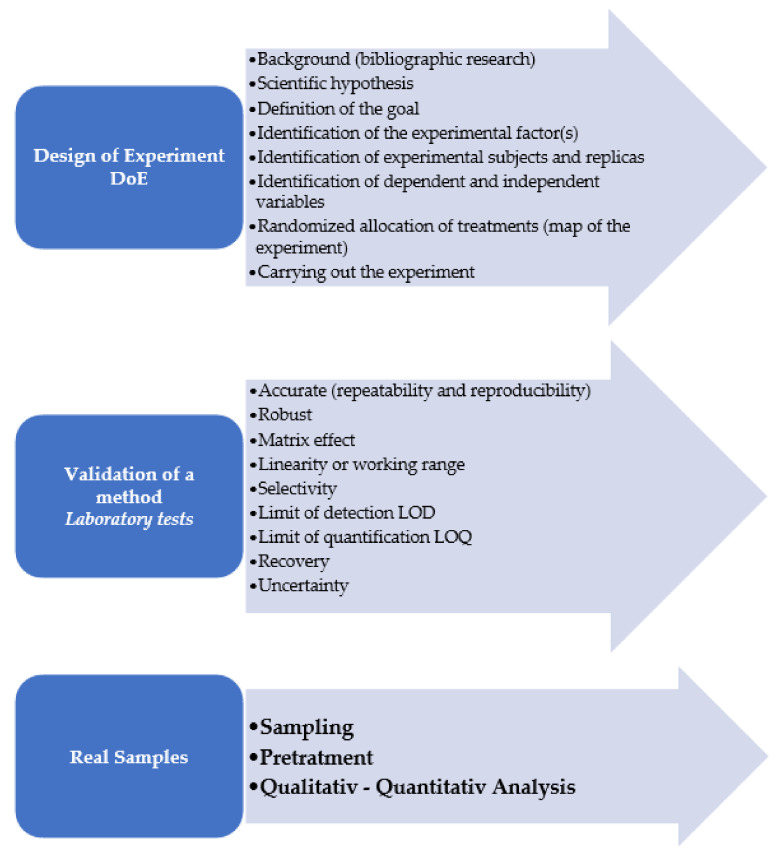
Outline of the main steps that should include the analysis of emerging compounds such as nanoplastics.

Further studies are recommended to develop protocols useful to increase the current knowledge regarding the spatial and temporal distribution of MPs and NPs. It is necessary to establish and maintain long-term monitoring programs using standardized methods that include a DoE to control changes in the spread of MPs and NPs diffusion in the environment over time considering the behavior of ENMs (Figure 3).

## Figures and Tables

**Table 1:** List of different techniques for NPs and MPs analysis.

Technique	Size Range	About	Advantage	Disadvantage
FTIR	>10 μm	Polymer identification	Easy, no sample preparation Non destructive	Time consuming
ATR-FTIR	>500 μm	Polymer identification	Easy, no sample preparation Non destructive	Time consuming
micro-FTIR	<20 μm	Polymer identification	Easy, no sample preparation Non destructive	Time consuming
IR-NMR		Polymer degradation	Identification of functional groups	Sample preparation Expensive
Raman	>10 μm	Polymer identification	Non destructive	Time consuming
Micro-Raman	>1 μm	Polymer identification	High resolution	Fluorescence interference
TED-GC-MS		Polymer identification	Sample can be heterogeneous No sample preparation	Destructive analysis
Optical microscopy		Size, color, morphology	Easy	No polymer characterization
SEM		Size, color, morphology	High resolution of images	Sample preparation
TEM	<0.1 nm		High resolution	Sample preparation Method in the early stage, no NPs have been detected
TGA-DSC		Polymer and additives identification	Easy	Destructive analysis
HPLC		Polymer and additives identification	Highly sensitive	Long sample preparation
Stereo microscope	>100 μm	Shape, size, and colors	Fast	No polymer characterization
Atomic force microscopy	>0.3 nm	Surface analysis	High resolution Polymer blend characterization	Sample damage Combination with other technique is needed
Fluorescence microscopy			Useful for transparent MNPs	Interference Use dye Pre-treatment
Atomic force microscopy	0.3 nm	Surface structure	High resolution	Possible contamination during the sample manipulation
Pyrolysis GC-MS		Polymer identification	Highly sensitive in combination with TGA	Destructive analysis
Cytometry	>200 nm	Quantification in liquid sample	Fast	Interference Use dye Pre-treatment

## Data Availability

Not applicable.

## Supplementary Materials

The following are available online at www.mdpi.com/article/10.3390/polym13213658/s1, Figure S1: Trend of publications on Microplastics from 2010 to August 2021. Comparison of studies focused on the water matrix compared to all the other matrices. Data collected from Scopus., Table S1: Development of techniques of different steps on different matrices samples.
