# Supplementary material for: Searching Nanoplastics: From Sampling to Sample Processing
Source: Polymers (Basel). 2021 Oct 23;13(21):3658. doi: 10.3390/polym13213658 (PMC8588424; doi:10.3390/polym13213658)
Supplement: Supplementary file 1 [file polymers-13-03658-s001.zip › polymers-1398341-supplementary.pdf]

polymers

MDPI

Supplementary Materials

Searching Nanoplastics: From Sampling to Sample Processing

Marina Cerasa [1], Simona Teodori [2] and Loris Pietrelli [2,*]

[1] National Research Council of Italy-Institute of Atmospheric Pollution Research (CNR-IIA), Via Salaria km 29,300, 00015 Monterotondo (RM), Italy; marina.cerasa@iia.cnr.it
[2] Chemistry Department, Sapienza University of Rome, Piazzale Aldo Moro, 00185 Rome, Italy; simona.teodori@uniroma1.it
* Correspondence: loris.pietrelli@uniroma1.it

Literature search

We conducted a comprehensive literature survey between July – August 2021 using accessible online databases such as Web of Science, Scopus, ScienceDirect and Scholar Google.

Citation: Cerasa, M.; Teodori, S.; Pietrelli, L. Searching Nanoplastics: From Sampling to Sample Processing. *Polymers* **2021**, *13*, 3658. https://doi.org/10.3390/polym13213658

Academic Editor(s): Leon Chernin

Received: 12 September 2021
Accepted: 20 October 2021
Published: 23 October 2021

Publisher's Note: MDPI stays neutral with regard to jurisdictional claims in published maps and institutional affiliations.

Copyright: © 2021 by the authors. Licensee MDPI, Basel, Switzerland. This article is an open access article distributed under the terms and conditions of the Creative Commons Attribution (CC BY) license (http://creativecommons.org/licenses/by/4.0/).

Table S1. Development of techniques of different steps on different matrices samples.

Sample/Matrix	Polymer	Pretreatment	Analyses	Refs.
seawater	PET, PS, PE, PVC	Ultrafiltration	DLS and pyrolysis coupled with GC-MS	[1]
seawater	PS, PE, PMMA and PA6	Raman Tweezer	Raman images	[2]
River water	PE, PS, PP	thermogravimetric furnace (TGA), PDMS solid-phase adsorbent	TDU- GC–MS	[3]
RIVER, SEA, WWTP influent and effluent	Spiked PS and PMMA	Triton X-45 - cloud-point extraction (CPE)	Py-GC-MS	[4]
River water and fish	Spiked PS and PET	alkaline digestion (KOH)<br>Membrane filtration	MALDI-TOF MS	[5]
fish	Spiked PS	acid and enzymatic digestion (proteinase K)<br>asymmetric flow field-flow fractionation (AF4)	multi-angle light scattering (MALS)	[6]
fish	PE, PS Spiked	hydrogen peroxide solution	Fourier transformed infrared spectrometry (FT-IR)	[7]
fish	cellulose acetate (CA), HDPE & LDPE, polylauryllactam (PA-12), polycaprolactam (PA-6), polycarbonate (PC), PET, PMMA, PP, PS, crosslinked polystyrene (PSXL), PTFE, PUR, unplasticized polyvinyl chloride (uPVC), Expanded polystyrene (ePS)	alkaline digestion<br>filtration	Pyr-GC/MS and Raman micro-spectrometry	[8]
suspended particulate matter	wood plastic composite (WPC), PP, PE	-	TED-GC–MS	[9]

sediments	PE, PET, PVC, PP	Sediment Microplastic Isolation Unit (SMI) / zinc chloride ($ZnCl_2$) solution	attenuated total reflectance (ATR) FT-IR	[10]
marine sediments	Spiked HD-PE, PP, PVC, PS, PET	Fenton's reagent	carbon-sulfur analyzer	[11]
Snow, air, ice	PE, PET, PS, PP, PPC, PVC	shaken and filtration and Low-pressure evaporation/sublimation	TD-PTR-MS	[12] [13]
Air indoor Outdoor		ultracentrifugation	ICP-MS, MALDI-TOF-MS	[14]
Ambient air	PS, PMMA	hydrogen peroxide solution	SERS with Raman	[15]
Air indoor	PET, fibers	-	FT-IR, Pyr-GC–MS	[16]

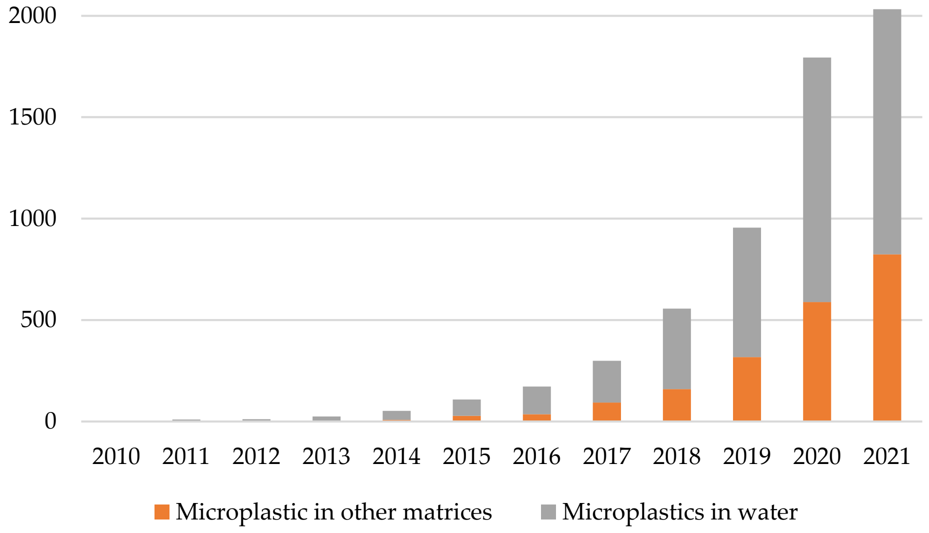

Figure S1 Trend of publications on Microplastics from 2010 to August 2021. Comparison of studies focused on the water matrix compared to all the other matrices. Data collected from Scopus.

1. Ter Halle, A.; Jeanneau, L.; Martignac, M.; Jardé, E.; Pedrono, B.; Brach, L.; Gigault, J. Nanoplastic in the North Atlantic Subtropical Gyre. *Environ. Sci. Technol.* **2017**, *51*, 13689–13697, doi:10.1021/acs.est.7b03667.
2. Gillibert, R.; Balakrishnan, G.; Deshoules, Q.; Tardivel, M.; Magazzù, A.; Donato, M.G.; Maragò, O.M.; Chapelle, M.L. de La; Colas, F.; Lagarde, F.; et al. Raman Tweezers for Small Microplastics and Nanoplastics Identification in Seawater. *Environ. Sci. Technol.* **2019**, *53*, 9003–9013, doi:10.1021/ACS.EST.9B03105.
3. Cunliffe, M.; Engel, A.; Frka, S.; Gašparović, B.Ž.; Guitart, C.; Murrell, J.C.; Salter, M.; Stolle, C.; Upstill-Goddard, R.; Wurl, O. Sea surface microlayers: A unified physicochemical and biological perspective of the air-ocean interface. *Prog. Oceanogr.* **2013**, *109*, 104–116, doi:10.1016/j.pocean.2012.08.004.
4. Zhou, X.; Hao, L.; Wang, H.; Li, Y.; Liu, J. Cloud-Point Extraction Combined with Thermal Degradation for Nanoplastic Analysis Using Pyrolysis Gas Chromatography–Mass Spectrometry. *Anal. Chem.* **2018**, *91*, 1785–1790, doi:10.1021/ACS.ANALCHEM.8B04729.
5. Lin, Y.; Huang, X.; Liu, Q.; Lin, Z.; Jiang, G. Thermal fragmentation enhanced identification and quantification of polystyrene micro/nanoplastics in complex media. *Talanta* **2020**, *208*, 120478, doi:10.1016/j.talanta.2019.120478.
6. Correia, M.; Loeschner, K. Detection of nanoplastics in food by asymmetric flow field-flow fractionation coupled to multi-angle light scattering: possibilities, challenges and analytical limitations. *Anal. Bioanal. Chem.* **2018**, *410*, 5603–5615, doi:10.1007/s00216-018-0919-8.
7. Avio, C.G.; Gorbi, S.; Regoli, F. Experimental development of a new protocol for extraction and characterization of microplastics in fish tissues: First observations in commercial species from Adriatic Sea. *Mar. Environ. Res.* **2015**, *111*, 18–26, doi:10.1016/j.marenvres.2015.06.014.
8. Dehaut, A.; Cassone, A.L.; Frère, L.; Hermabessiere, L.; Himber, C.; Rinnert, E.; Rivière, G.; Lambert, C.; Soudant, P.; Huvet, A.; et al. Microplastics in seafood: Benchmark protocol for their extraction and characterization. *Environ. Pollut.* **2016**, *215*, 223–233, doi:10.1016/j.envpol.2016.05.018.
9. Duemichen, E.; Eisentraut, P.; Celina, M.; Braun, U. Automated thermal extraction-desorption gas chromatography mass spectrometry: A multifunctional tool for comprehensive characterization of polymers and their degradation products. *J. Chromatogr. A* **2019**, *1592*, 133–142, doi:10.1016/j.chroma.2019.01.033.
10. Díaz-Jaramillo, M.; Islas, M.S.; Gonzalez, M. Spatial distribution patterns and identification of microplastics on intertidal sediments from urban and semi-natural SW Atlantic estuaries. *Environ. Pollut.* **2021**, *273*, 116398, doi:10.1016/j.envpol.2020.116398.
11. Lin, J.; Xu, X.-P.; Yue, B.-Y.; Li, Y.; Zhou, Q.-Z.; Xu, X.-M.; Liu, J.-Z.; Wang, Q.-Q.; Wang, J.-H. A novel thermoanalytical method for quantifying microplastics in marine sediments. *Sci. Total Environ.* **2021**, *760*, 144316, doi:10.1016/j.scitotenv.2020.144316.
12. Materić, D.; Ludewig, E.; Brunner, D.; Röckmann, T.; Holzinger, R. Nanoplastics transport to the remote, high-altitude Alps. *Environ. Pollut.* **2021**, *288*, 117697, doi:10.1016/j.envpol.2021.117697.
13. Materić, D.; Kasper-Giebl, A.; Kau, D.; Anten, M.; Greilinger, M.; Ludewig, E.; Van Sebille, E.; Röckmann, T.; Holzinger, R. Micro-and Nanoplastics in Alpine Snow: A New Method for Chemical Identification and (Semi)Quantification in the Nanogram Range. *Environ. Sci. Technol.* **2020**, *54*, 2353–2359, doi:10.1021/acs.est.9b07540.
14. Velimirovic, M.; Tirez, K.; Verstraelen, S.; Frijns, E.; Remy, S.; Koppen, G.; Rotander, A.; Bolea-Fernandez, E.; Vanhaecke, F. Mass spectrometry as a powerful analytical tool for the characterization of indoor airborne microplastics and nanoplastics. *J. Anal. At. Spectrom.* **2021**, *36*, 695–705, doi:10.1039/d1ja00036e.
15. Xu, G.; Cheng, H.; Jones, R.; Feng, Y.; Gong, K.; Li, K.; Fang, X.; Tahir, M.A.; Valev, V.K.; Zhang, L.

Surface-Enhanced Raman Spectroscopy Facilitates the Detection of Microplastics <1 μm in the Environment. *Environ. Sci. Technol.* **2020**, *54*, 15594–15603, doi:10.1021/acs.est.0c02317.

16. O'Brien, S.; Okoffo, E.D.; O'Brien, J.W.; Ribeiro, F.; Wang, X.; Wright, S.L.; Samanipour, S.; Rauert, C.; Toapanta, T.Y.A.; Albarracin, R.; et al. Airborne emissions of microplastic fibres from domestic laundry dryers. *Sci. Total Environ.* **2020**, *747*, 141175, doi:10.1016/j.scitotenv.2020.141175.
